# Clinical Application of Individualized Pulmonary Bi-Orifice for the Reconstruction of Right Ventricular Outflow Tract in Tetralogy of Fallot

**DOI:** 10.3389/fcvm.2021.772198

**Published:** 2021-11-26

**Authors:** Ming Wu, Chengming Fan, Jian Liu, Chukwuemeka Daniel Iroegbu, Wangping Chen, Peng Huang, Mi Tang, Xun Wu, Chunle Wang, Kun Xiang, Wenwu Zhou, Jinfu Yang

**Affiliations:** ^1^Department of Cardiovascular Surgery, The Second Xiangya Hospital, Central South University, Changsha, China; ^2^Department of the Cardiovascular Surgery, The Hunan Provincial People's Hospital, Changsha, China; ^3^Department of Cardiothoracic Surgery, Hunan Children's Hospital, Changsha, China

**Keywords:** congenital heart disease, Tetralogy of Fallot, right ventricular outflow tract reconstruction, bi-orifice, pulmonary valve

## Abstract

**Objective:** The study aims to establish a new method in the Tetralogy of Fallot (ToF) called the pulmonary valve bi-orifice method (pulmonary annular sparing with an individualized autologous pericardial patch; thus, two orifices are formed at the level of the pulmonary valve annulus) to reconstruct the right ventricular outflow tract (RVOT).

**Methods:** A retrospective analysis of 128 TOF patients from October 2009 to June 2018 with severe pulmonary valve dysplasia who underwent transvalvular annular patch (TAP) procedure (control group) or an individualized pulmonary valve bi-orifice procedure (observation group) were studied. The RVOT for each patient in the observation group was individually reconstructed per the patient's weight and the size of the autologous pulmonary valve using the bi-orifice method; however, increasing the cross-sectional area of the pulmonary valve annulus without destroying its integrity. The result was then compared to the control group, where TAP procedures were applied to evaluate the short to mid-term outcome(s). An *in vitro* simulation test was used to verify the anti-regurgitation mechanism of the new method.

**Results:** The *in vitro* simulation test indicated that the anti-regurgitation mechanism was completed by the pericardial patch and the autologous pulmonary valve movement toward each other. Thus, for clinical applications, patients in both groups were compared. The results showed no significant differences in cardiopulmonary bypass and aortic cross-clamp time, mechanical ventilation, and ICU and post-operative residence between the two groups.

During the follow-up period (3- to 12-years), 14 patients in the observation group had mild regurgitation after surgery (22.2%), while 10 patients had moderate pulmonary regurgitation (15.8%) with no right ventricular (RV) dilation. On the other hand, 22 patients (39.6%) had moderate to severe regurgitation in the control group, while left pulmonary artery stenosis occurred in one patient. In the control group, six patients (9.2%) with severe RV dilation were reoperated.

**Conclusion:** Individualized pulmonary valve bi-orifice procedure is a safe and excellent method for reconstructing RVOT in ToF.

## Background

Tetralogy of Fallot (ToF) is the most prevalent cyanotic congenital heart disease in pediatric cardiac surgery, accounting for 12–14% of all congenital heart diseases, with surgery currently the only way of treating ToF. The surgical process for this congenital anomaly typically includes the reconstruction of the right ventricular outflow tract (RVOT), given that ToF is usually accompanied by dysplasia of the pulmonary valve and its annulus. The first adopted surgical procedure have always included incision of the pulmonary valve annulus and a transannular patch (TAP) to widen the dysplastic pulmonary valve (1), and achieved short-term favorable results (2).

Though stenosis in the RVOT is alleviated with the procedure; however, subsequent severe pulmonary regurgitation is encountered few years after the procedure, even worse than the initial residual stenosis (3). Interestingly, procedures such as the valve-spring technique have been used to improve the setbacks. However, favorable surgical outcomes with these techniques, especially in severely dysplastic pulmonary valves, are lacking. Therefore, reconstructing the RVOT is crucial for a favorable long-term surgical outcome. Thus, we developed a new procedure to partially separate the pulmonary annulus from the pulmonary arterial wall, preserving its integrity and using a pericardial patch to widen the RVOT.

The method used in the study herein has since been applied in our institution from 2009 to decrease pulmonary regurgitation. The present study also aimed to individualize the procedure to achieve better physiological blood flow.

## Methods

### Clinical Data

The retrospective study analyzed clinical data of 128 TOF patients who underwent TAP (control group) or an individualized pulmonary valve bi-orifice (observation group) procedures between October 2009 and June 2018 from three heart centers: The Second Xiangya Hospital of Central South University, Hunan Children's Hospital, and Hunan Provincial People's Hospital. There were 63 patients (mean age 14.2 ± 6.7 months) in the observation group and 65 patients (mean age 12.6 ± 4.8 months) in the control group (Table 1).

**Table 1:** Preoperative data from the two groups.

	**Observation group**	**Control group**	***P-*value**
Gender (male/female)	34/29	30/35	
Age (month)	14.2 ± 6.7	12.6 ± 4.8	0.113
Weight (kg)	9.32 ± 1.63	9.12 ± 1.16	0.423
oxygen saturation	80.99 ± 3.11%	80.34 ± 3.44%	0.344
Mean RVOT gradient	67.97 ± 9.86	67.32 ± 9.03	0.7
McGoon index	1.38 ± 0.07	1.36 ± 0.06	0.116
Z-value of pulmonary annulus	−2.58 ± 0.30	−2.61 ± 0.26	0.502

*RVOT, right ventricular outflow tract*.

The patients in the study were all cyanotic. The oxygen saturation in their upper and lower limbs ranged from 73 to 87% at rest. Echocardiography confirmed the diagnosis of TOF with hypertrophy of the right atrium and ventricle, massive ventricular septal defect (VSD), aortic straddle, and stenosis in the RVOT. Computed cardiac tomography was used to identify the diameter of the pulmonary annulus, main pulmonary artery, and its branches (Table 1).

The present study enrolled the TOF patients suitable for the TAP technique and randomly divided the selected patients into control and observation groups. It should be noted that patients were not included in the study if the selected ToF patient had other severe congenital cardiac malformations, a McGoon index <1.2, and a pulmonary annular *Z*-value >−2.

Consents from all patients and the Institutional Research Ethics Committee were obtained beforehand. Preoperative written and informed consent from all participants and their families was obtained.

### Surgical Process

**Figure 1:**
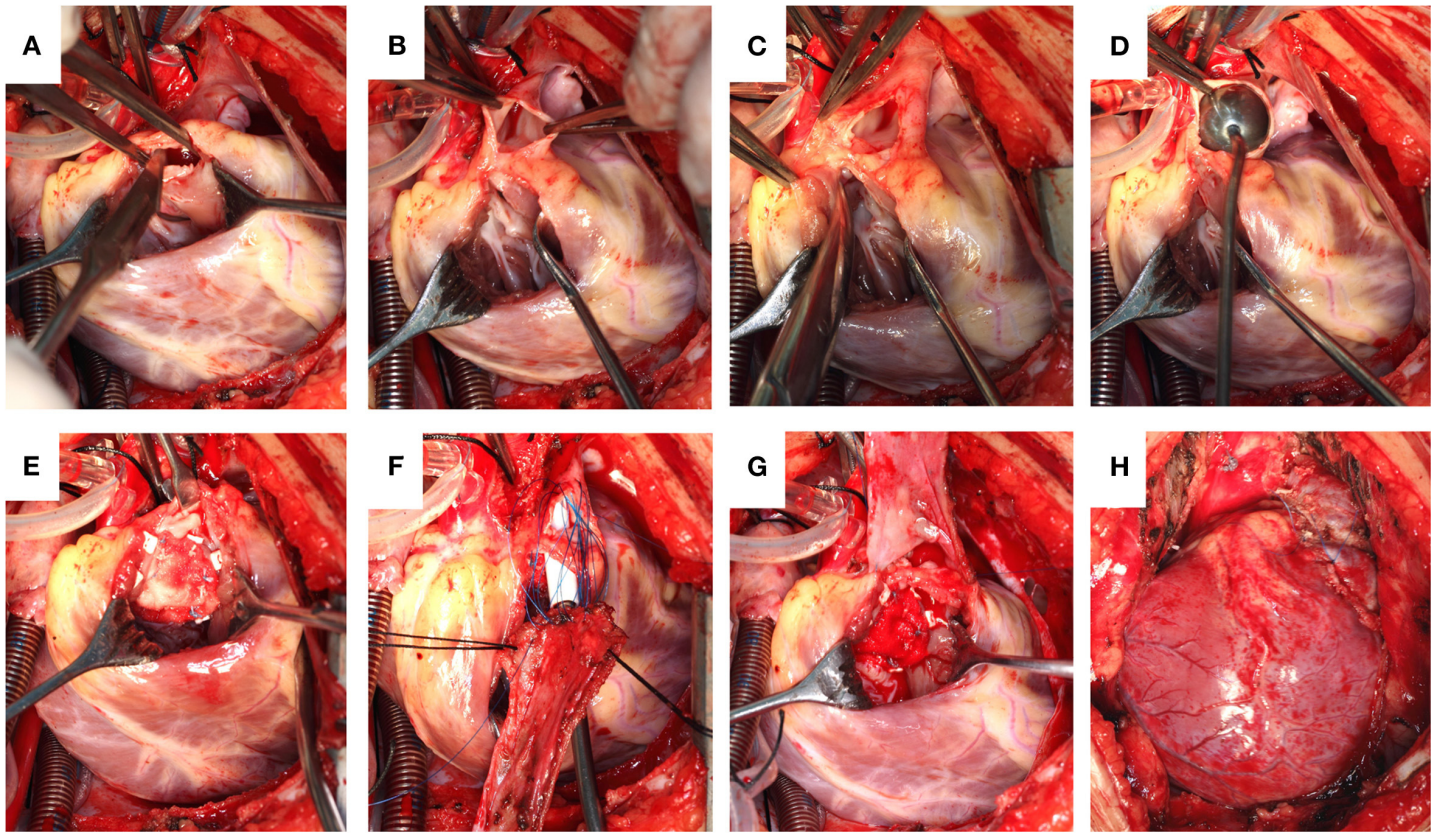
**(A)** Pulmonary valve commissurotomy performed via a right ventricular incision. **(B)** The pulmonary artery incised, reserving the integrity of the pulmonary valve annulus. **(C)** Partially separated pulmonary valve annulus from the pulmonary wall. **(D)** Size of the distal pulmonary artery explored. **(E)** Repaired ventricular septal defect. **(F)** A piece of the autologous pericardium used to widen the RVOT with a sucker held deep into the pulmonary valve annulus to reveal the first orifice. **(G)** A soft autologous pericardial patch sutured continuously from the distal pulmonary incision to the RV. **(H)** Completed reconstruction.

Longitudinal sternotomy and a cardiopulmonary bypass procedure under moderate hypothermia (28–30°C) were performed for the patients following satisfactory results of the *in vitro* tests. After bypass, HTK cardioplegia was injected via the aortic root, and an ice flush was applied on the heart surface to protect the myocardium following arrest. A longitudinal right ventricular (RV) incision was made, and a pulmonary valve commissurotomy was performed. After that, the front pulmonary artery wall was longitudinally incised along the midline, terminating at the valve annulus. The partial pulmonary annulus was then separated from the arterial wall on both sides toward the valve junction, preserving the pulmonary annular integrity. Following the VSD repair, a soft autologous pericardial patch was sutured continuously from the distal pulmonary incision to the RV (Figure 1). In the control group, a TAP was used.

**Figure 2:**
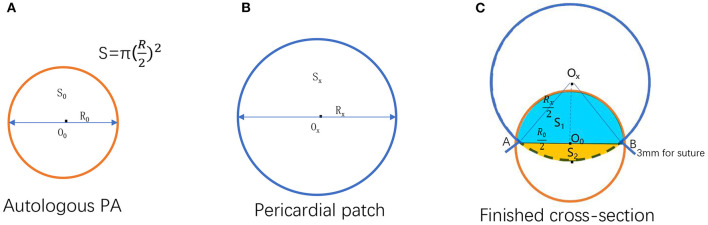
Illustration for formula deduction. **(A)** Autologous pulmonary artery cross-section. **(B)** A fictitious circle formed by the pericardial patch before the procedure. **(C)** The cross-section of the reconstructed pulmonary valve annulus that combined the pericardial patch and the autologous pulmonary artery after the operation [O_0_, O_x_: The center of the circle formed by autologous pulmonary valve annulus and pericardial patch, respectively; The area and the diameter of the autologous pulmonary valve annulus, respectively; the area and the diameter of the circle formed by uncorrected pericardial patch, respectively; The area and the diameter of the circle formed by the corrected pericardial patch, respectively. The overlapped part of the circle formed by the autologous pulmonary artery and pericardial patch after reconstruction; **(A,B)** Suture point of the autologous pulmonary artery and pericardial patch].

Notably, there are significant individual differences with RVOT in ToF, precisely its development, including the annulus and valve leaflets. For individualized reconstruction of the RVOT, an autologous pericardial patch of appropriate size was harvested using a formula. As shown in Figure 2A is the cross-section of the autologous pulmonary artery, while Figure 2B is a fictitious circle formed by the pericardial patch before the procedure. The two circles' total area equals the pulmonary annular cross-sectional area to be attained after reconstruction (*S*_*x*_ = *S*_*t*_-*S*_0_) ①. Per the formula of a circle, we can get $R_{x}=\sqrt{{R_{t}}^{2}-{R_{0}}^{2}}\;$ ②. Figure 2C is the cross-section that combines the autologous patch and the autologous pulmonary artery following reconstruction. When two circles are sutured together, as shown in Figure 2C, the actual area will be reduced by the overlapping part of both circles (*S*_1_ + *S*_2_, $S_{1}=\frac{1}{2}S_{0}$).

Therefore, the pericardium should be more significant to make up for the loss (overlapping part) i.e., ($\frac{1}{2}S_{0}+S_{2})$,. Use *S*_*xa*_ to represent the area of the circle formed by the corrected pericardial patch $\left(S_{xa}=S_{x}+\frac{1}{2}S_{0}+S_{2}\right)$. Substitute ① into this formula: *S*_*xa*_ = *S*_*t*_ − $S_{0}+\frac{1}{2}S_{0}+S_{2}=S_{t}$ − $\frac{1}{2}S_{0}+S_{2}$. Since *S*_2_ is usually very small and can be ignored for the convenience of calculation, it can be simplified as *S*_*xa*_ = *S*_*t*_ − $\frac{1}{2}S_{0}$; therefore, ${\;R}_{xa}=\sqrt{{R_{t}}^{2}-\frac{1}{2}{R_{0}}^{2}}\;$ ③. It can be seen from Figure 2C that L is a part of the combined circle, which equals the circumference of the whole circle minus the length of the arc AB. In addition, there is a patch loss of about 3 mm for sutures on each side. Thus, it can be deduced that L = π*R*_*xa*_ − $\frac{∠AOxB}{360}\pi R_{xa}+$ 6 mm = (1 − $\frac{∠AOxB}{360}$) π*R*_*xa*_ + 6 mm ④. As shown in Figure 2C, $\sin\frac{1}{2}∠AOxB\;=\;\frac{Ro/2\;}{Rx/2}\;=$ $\frac{Ro}{Rx}\;$ per the geometric calculation. By extrapolation, ∠AOxB = 2 arc $\sin\frac{R_{0}}{R_{X}}\;$ ⑤. Finally, it can be derived from ③, ④, ⑤ that L = π (1$-\frac{2\text{arc}\sin\frac{R_{0}}{\sqrt{{R_{t}}^{2}-{R_{0}}^{2}}}}{360}$)$\sqrt{{R_{t}}^{2}-\frac{1}{2}{R_{0}}^{2}}$ + 6.

### The *in vitro* Simulation Test

An *in vitro* test was used to verify the feasibility of the surgical method further. Two primary materials, namely a porcine heart and a bovine pericardium patch from Alance Medical, were used to create 10 models. The RV cone, pulmonary valve, and the main pulmonary artery of the porcine heart were harvested. The conical muscles in the RV chamber under the valve were all directly cut off, reserving a significant enough edge for suture. The front pulmonary artery wall was longitudinally incised along the midline to the valve annulus, while the pulmonary annulus was carefully separated from the arterial wall toward both sides, close to the valve junction.

A bovine pericardial patch was continuously sutured to the main pulmonary artery to widen the artery and the pulmonary annulus. The new pulmonary annulus comprises the bovine pericardial patch and the separated annulus, retaining the pulmonary valve leaflets. Both ends of the new pulmonary artery were fixed on a stent for the test. The models were applied on a pulse duplicator (Vivitro Labs, Canada), where the cooperative anti-regurgitation mechanism of the pulmonary valve and the pericardial patch was observed. The test parameters were mean pulmonary blood pressure 20 mmHg, pump stroke 97.2 ml, heart rate 70 bpm, and systolic duration 50% (Supplementary Video 1).

### Follow-Up

Patients were post-operatively followed up at 1 month, 6 months, and then every year after discharge. The pulmonary regurgitation jet width/annular ratio detected by transthoracic echocardiography was used to assess PR severity after RVOT reconstruction. Ratios <25% were defined as mild, while 25–50% as moderate, and >50% as severe. Severe pulmonary regurgitation accompanied by continuous RV dilation will be considered for pulmonary valve replacement.

### Statistical Methods

All statistical calculations were performed using the Statistical Product and Service Solutions 14.0 software (SPSS Institute). If data conformed to normal distribution and homogeneity of variance, the data of patients in the hospital, including baseline and clinical data were analyzed by independent samples *t*-test. Follow-up data were analyzed by Gehan-Breslow-Wilcoxon test and Log-rank test. Data are expressed as mean ± SE. The critical alpha level for these analyses was set at *p* < 0.05.

## Results

### Clinical Results

There was no significant difference between the two groups in age, weight, blood oxygen saturation, McGoon value, and pulmonary annular *Z*-value (Table 1). There was no death. Post-operative echocardiography showed that the two groups had no residual ventricular septal shunt, and all deformities were satisfactorily repaired. There was no third-degree atrioventricular block, and the patients' limb oxygen saturation was ≥95% after the operation. Also, there were no significant differences in cardiopulmonary bypass and aortic cross-clamp time, mechanical ventilation, and ICU and post-operative residence between the two groups.

**Figure 3:**
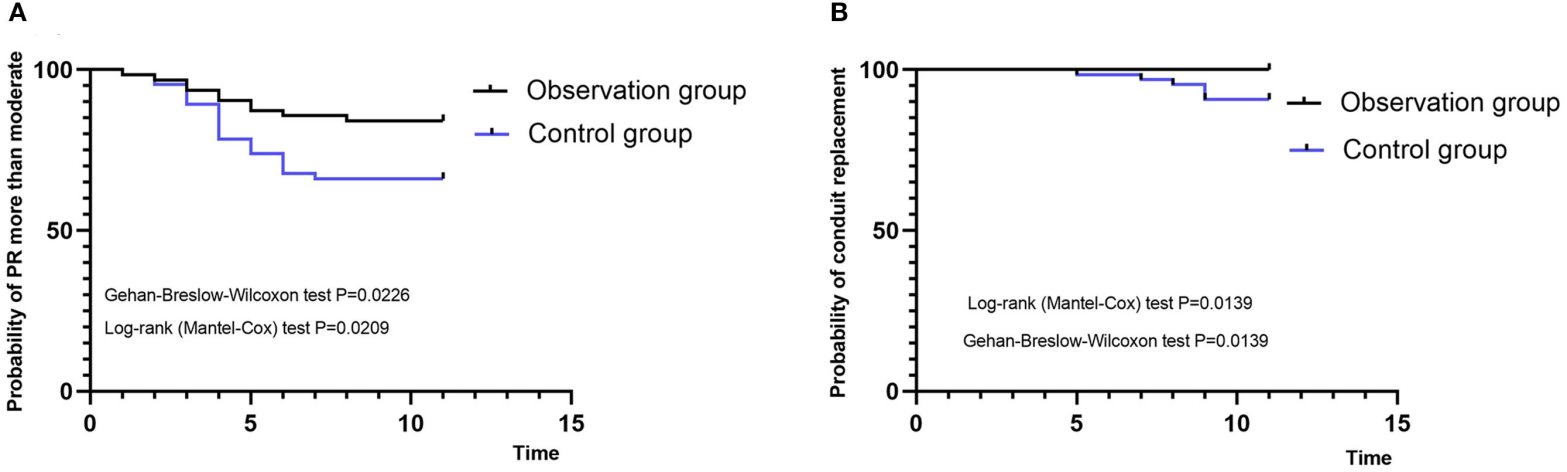
Kaplan-Meier curves. **(A)** Probability of moderate and serious pulmonary regurgitation. **(B)** Probability of reoperation. PR, pulmonary regurgitation.

During the follow-up period (3- to 12-years, Figure 3), no patient had severe regurgitation in the observation group. However, 14 patients in the observation group had mild regurgitation after surgery (22.2%). Though moderate regurgitation occurred in 10 patients (15.8%), no patient presented with RV dilation. In the control group, 22 cases (33.8%) had moderate to severe regurgitation, and left pulmonary artery stenosis occurred in one patient, while others had mild regurgitation. In the control group, six patients (9.2%) were reoperated following severe dilation of the RV (Table 2).

**Table 2:** Post-operative and follow-up data from the two groups.

	**Observation group**	**Control group**	**Value**
Cardiopulmonary bypass time (min)	97.86 ± 9.46	97.62 ± 8.49	0.879
Aortic cross-clamp time (min)	68.57 ± 8.90	66.11 ± 7.79	0.098
Mechanical ventilation time (h)	67.71 ± 12.08	71.32 ± 12.02	0.093
ICU residence time (day)	6.57 ± 1.05	6.81 ± 1.17	0.232
Post-operative residence time (day)	13.40 ± 2.83	13.80 ± 2.87	0.425
Moderate and serious VR	10	22	0.0226^*^
Moderate VR	10	13	
Serious VR	0	9	
Conduit replacement	0	6	0.0139^*^

* *Gehan-Breslow-Wilcoxon test; ICU, intensive care unit; VR, valvular regurgitation*.

### The *in vitro* Simulation Test

**Figure 4:**
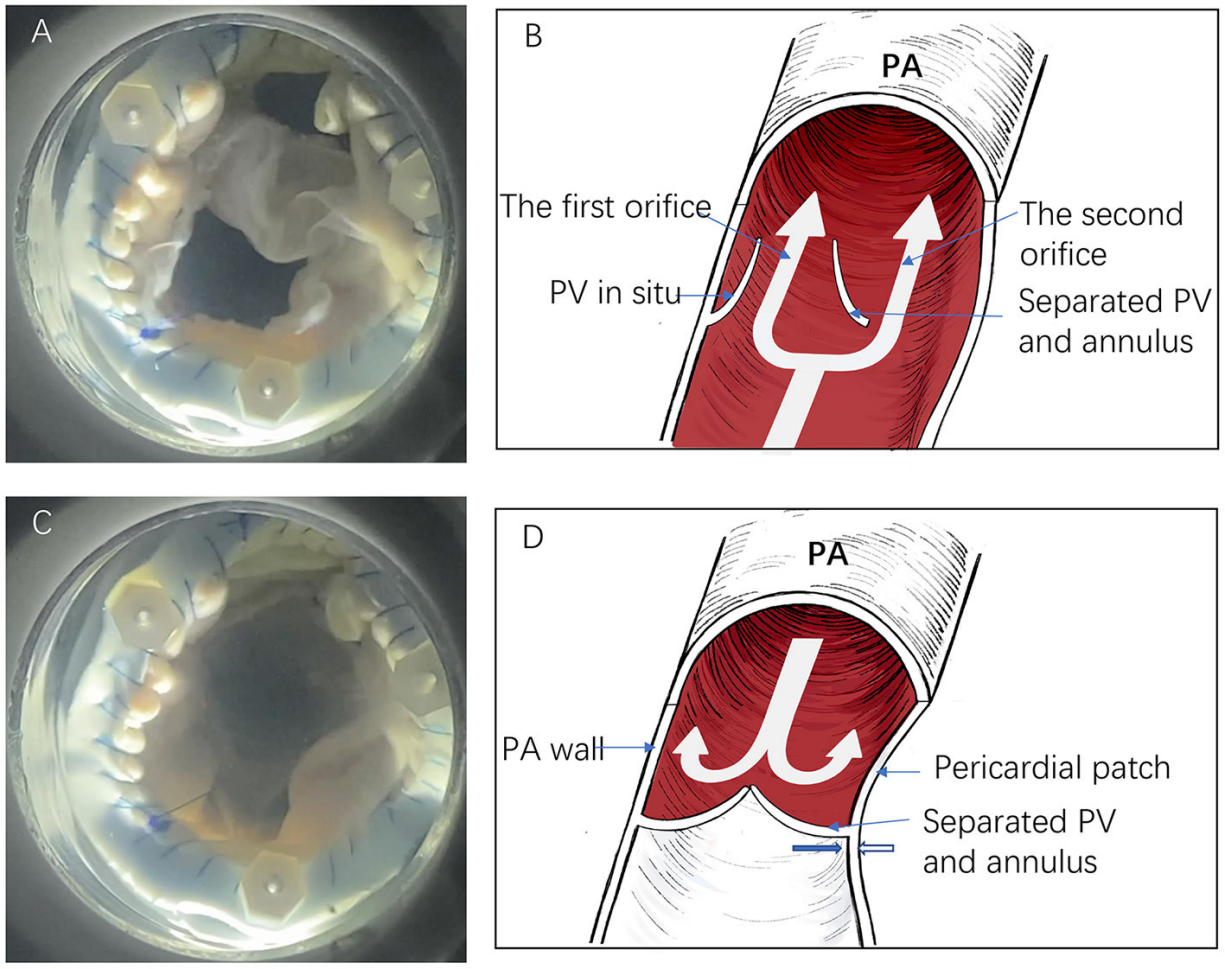
The *in vitro* simulation test and descriptive figure illustrating anti-regurgitative mechanism. **(A,B)** The forward blood flow passes via the two orifices during the systole. **(C,D)** The second orifice closes following a negative pressure effect (Bernoulli effect) from the right ventricle. The thick blue and white arrows indicate the separated pulmonary valve and pericardial patch direction, respectively (PV, pulmonary valve; PA, pulmonary artery).

During systole, the forward direction of the blood flow forces the pulmonary valve open, thus causing an outward expansion of the pulmonary artery, providing an easy flow of blood via the two orifices. During diastole, the pericardial patch and the autologous pulmonary valve move toward each other, closing the valveless space between the pericardial patch and the pulmonary valve (Figure 4; Supplementary Video 1). The forward flow volume and the leakage volume were 86.07 ± 1.30 and −0.72 ± 0.86 ml, respectively. The forward energy loss through active position and the leakage were 275.3 ± 3.20 and 2.2 ± 1.30 mJ, respectively (Supplementary Table 1).

### Discussion and Conclusion

The TAP surgical technique was the earliest and presently the most widely used surgical procedure adopted to achieve favorable short-term results for severe pulmonary valve dysplasia patients. The procedure has always involved reconstructing the RVOT using a TAP with a damaged pulmonary annulus. Pulmonary regurgitation causes RV volume overload and insufficiency, intractable arrhythmia, and sudden death (3–7). Hence, additional surgical intervention is required for such patients to effectively correct pulmonary valve regurgitation and improve prognosis (8).

Notably, the concept of RVOT reconstruction in ToF surgery for the past 20-years has undergone a subversive change. In the past, attention was paid to the complete alleviation of RVOT obstruction; currently, surgeons are willing to preserve the function of the autologous pulmonary valve, even though there is residual RVOT obstruction (9–11). Several studies currently focus on the valve-sparing strategy (9–14) and supplementary measures such as additional aggressive commissurotomy (15), leaflet delamination (16), and balloon dilatation valvuloplasty under direct vision (16, 17) to enlarge the pulmonary valve orifice. Nonetheless, residual obstruction and pulmonary regurgitation are still risks for re-intervention, which limit its use (scope) and requires vast experience and technical know-how.

On the other hand, the borderline from valve-sparing to TAP and avoiding an unacceptable residual obstruction remains challenging to establish. In addition, there are methods of sewing 1–2 artificial valve leaflets with transvalvular patches made of autologous pericardial sheets and PTFE to decrease pulmonary valve regurgitation (18–21). Nath et al. (22) also reported using biological materials such as pulmonary homograft monocusp to reconstruct the RVOT; however, it is prone to calcification and tissue structure degradation.

**Figure 5:**
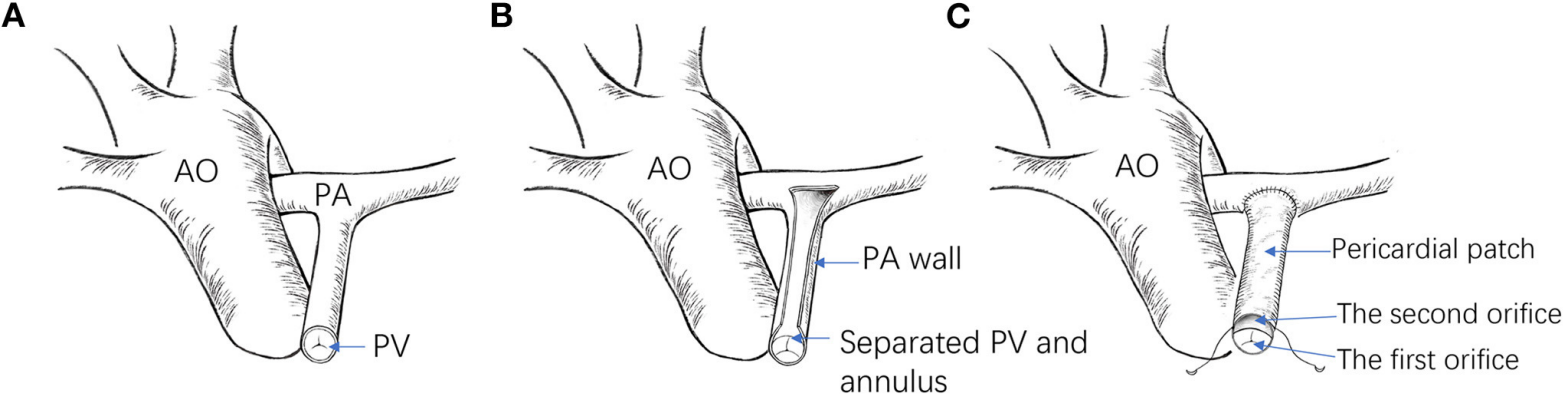
**(A)** Illustration of the RVOT before reconstruction. **(B)** The pulmonary artery is incised open, and the partial pulmonary annulus is separated from the pulmonary wall, reserving its integrity. **(C)** A pericardial patch is used to widen the RVOT (AO, aorta; PA, pulmonary artery; PV, pulmonary valve).

For an ideal RVOT reconstruction, the pulmonary stenosis should be corrected without pulmonary valve regurgitation. Here, we introduce a new method called pulmonary bi-orifice reconstruction (14). During the procedure, the partial pulmonary valve and annulus are separated from the pulmonary artery wall to preserve its integrity, while a pericardial patch is used to widen the RVOT to relieve the obstruction. Here, two orifices are formed at the level of the pulmonary valve annulus (Figure 5). Of significant importance, a pre-clinical animal study was designed and performed to verify the surgical method's feasibility further.

As is shown in the *in vitro* simulation test, the method formed an effective anti-regurgitation mechanism. During systole, the pressure of the forward blood flow passes via both orifices without obstruction. When the heart relaxes, the first orifice is closed because of the autologous pulmonary valve. Simultaneously, the RV develops a negative pressure effect (Bernoulli effect) that causes the pericardial patch to retract toward the separated pulmonary artery annulus while the separated autologous pulmonary annulus moves toward the patch. Hence, the valveless space between the pericardial patch and the pulmonary valve closes (Figure 4; Supplementary Video 1). Indeed, the anti-regurgitation mechanism undoubtedly benefits from low pulmonary artery pressure.

Interestingly, if the pericardial patch is too large, the second orifice is extensively large to form an effective anti-regurgitation effect. If the pericardial patch is too small, it is thus not enough to relieve the RVOT obstruction to meet physiological needs. We thus provided a summed-up formula to individualize the size of the pericardial patch per the size of the patient and the autologous pulmonary artery. Considering the growth potential of the pulmonary artery after reconstruction, the size of the reconstructed pulmonary artery aligns with physiological reality. Likewise, the pulmonary artery hemodynamic performance and the arterial wall-flow stress also physiologically align with stable hemodynamics. It, therefore, makes the pericardial patch align with the autologous pulmonary artery's growth and development; thus, avoiding twisting and obstruction of branch pulmonary arteries. Hence, a prospective study design and an ample sample size are pivotal to establishing the bi-orifice method's advantages and if; however, the outcomes are encouraging.

## Data Availability Statement

The original contributions presented in the study are included in the article/Supplementary Material, further inquiries can be directed to the corresponding author/s.

## Ethics Statement

The studies involving human participants were reviewed and approved by the Ethics Committee of the Second Xiangya Hospital of Central South University. Written informed consent to participate in this study was provided by the participants' legal guardian/next of kin.

## Author Contributions

MW and CF drafted the manuscript. WZ and JY designed the study. MW, JL, CI, WC, PH, MT, XW, CW, and KX revised the manuscript. MW was responsible for the collection of data or analysis. All authors read and approved the final manuscript.

## Funding

This work was supported by Hunan Provincial Health Commission (to JY); the key project of Science and Technology of Hunan Province (No. 2020SK53420 to JY); and the Science and Technology Innovation Program of Hunan Province (No. 2021RC2106 to CF).

## Conflict of Interest

The authors declare that the research was conducted in the absence of any commercial or financial relationships that could be construed as a potential conflict of interest.

## Publisher's Note

All claims expressed in this article are solely those of the authors and do not necessarily represent those of their affiliated organizations, or those of the publisher, the editors and the reviewers. Any product that may be evaluated in this article, or claim that may be made by its manufacturer, is not guaranteed or endorsed by the publisher.
