# Supplementary material for: Clinical Application of Individualized Pulmonary Bi-Orifice for the Reconstruction of Right Ventricular Outflow Tract in Tetralogy of Fallot
Source: Front Cardiovasc Med. 2021 Nov 26;8:772198. doi: 10.3389/fcvm.2021.772198 (PMC8661005; doi:10.3389/fcvm.2021.772198)
Supplement: Supplementary file 2 [file Table_1.docx]

**Supplemental Table 1** Postoperative data from the bi-orifice porcine pulmonary artery

| Parameter | Means±SD |
| --- | --- |
| Pressures（mmHg） | 15.1±0.10 |
| Transvalve Pressure through active position (mmHg) | 18.2±0.10 |
| Forward Flow Volume (ml) | 86.07±1.30 |
| Closing Volume (ml) | -4.59±0.57 |
| Leakage Volume (ml) | -0.72±0.86 |
| Energy Loss through active position, Forward (mJ) | 275.3±3.20 |
| Energy Loss through active position, Closing (mJ) | 8.1±1.10 |
| Energy Loss through active position, Leakage (mJ) | 2.2±1.30 |
